# Cancer in the offspring of radiation workers: an investigation of employment timing and a reanalysis using updated dose information

**DOI:** 10.1038/sj.bjc.6601273

**Published:** 2003-09-30

**Authors:** T Sorahan, R G E Haylock, C R Muirhead, K J Bunch, L J Kinlen, M P Little, G J Draper, G M Kendall, R J Lancashire, M A English

**Affiliations:** 1 Institute of Occupational Health, University of Birmingham, Edgbaston, Birmingham B15 2TT, UK; 2 National Radiological Protection Board, Chilton, Didcot, Oxon OX11 0RQ, UK; 3 Childhood Cancer Research Group, University of Oxford, 57 Woodstock Road, Oxford OX2 6HJ, UK; 4 CRC Cancer Epidemiology Research Group, Department of Public Health, University of Oxford, The Radcliffe Infirmary, Oxford OX2 6HE, UK; 5 Department of Epidemiology and Public Health, Imperial College Faculty of Medicine, St Mary's Campus, Norfolk Place, London W2 1PG, UK; 6 Department of Public Health and Epidemiology, University of Birmingham, Edgbaston, Birmingham B15 2TT, UK

**Keywords:** radiation, childhood cancer, occupational exposures

## Abstract

An earlier case–control study found no evidence of paternal preconceptional irradiation (PPI) as a cause of childhood leukaemia and non-Hodgkin's lymphoma (LNHL). Although fathers of children with LNHL were more likely to have been radiation workers, the risk was most marked in those with doses below the level of detection. The timing of paternal employment as a radiation worker has now been examined. The previously reported elevated risk of LNHL in the children of male radiation workers was limited to those whose fathers were still radiation workers at conception or whose employment also continued until diagnosis. Children whose fathers stopped radiation work prior to their conception were found to have no excess risk of LNHL. It was not possible to distinguish between the risks associated with paternal radiation work at conception and at the time of diagnosis. A reanalysis of the original study hypothesis incorporating updated dosimetric information gave similar results to those obtained previously. In particular, the risks of LNHL did not show an association with radiation doses received by the father before conception. It seems likely that the increased risk of LNHL among the children of male radiation workers is associated with an increased exposure to some infective agent consequent on high levels of population mixing.

A case–control study of leukaemia and lymphoma cases occurring between 1950 and 1985 among young persons (under 25 years of age) born and diagnosed in West Cumbria (Gardner *et al*, 1990; Gardner, 1992) found that paternal preconceptional exposures to external sources of whole–body ionising radiation during employment at the Sellafield nuclear installation were associated with a raised incidence of childhood leukaemia and non-Hodgkin's lymphoma (LNHL) in offspring. No similar associations were found for Hodgkin's disease.

Following the study of Gardner *et al* (1990), several studies examined the hypothesis of an association between childhood LNHL and paternal preconceptional irradiation (PPI). Among the largest of these was the Record Linkage Study (Draper *et al*, 1997a,Draper *et al*, 1997b). After excluding cases studied by Gardner *et al* (1990), there was no statistically significant increase in risk with increasing dose, for any of the exposure periods studied; indeed, the raised rate of childhood LNHL was mostly marked in the offspring of fathers with doses below the level of detection. The authors concluded that the study findings did not support the hypothesis that PPI is a cause of childhood LNHL. Fathers of children with LNHL were, however, significantly more likely than fathers of controls to have been radiation workers (relative risk 1.77, 95% confidence interval (CI) 1.05–3.03). It was postulated that this elevated risk might be a chance finding or result from exposure to infective or other agents.

There is evidence from studies testing the infective hypothesis of population mixing that transmission of infection by adults in such situations has a role in childhood leukaemia (Kinlen *et al*, 1993b; Kinlen, 1995; Stiller and Boyle, 1996). If cancer risk were associated with paternal exposure to an infective agent during the child's infancy, then paternal employment involving such exposure in the first few years of the child's life may be more important than preconceptional employment.

This paper reports analyses that examine the relevance of the timing of parental employment as a radiation worker (here defined as all types of employment with an organisation participating in the National Registry for Radiation Workers (NRRW)) in relation to the conception of the child. In addition, updated information on radiation dose is now available for some of the workers and a reanalysis using these data is presented.

## MATERIALS AND METHODS

The study methodology is described in detail elsewhere (Draper *et al*, 1997a,Draper *et al*, 1997b). Briefly, three sources of information about childhood tumours were used: namely, the National Registry of Childhood Tumours (NRCT) (Stiller *et al*, 1995), the Oxford Survey of Childhood Cancers (OSCC) (Stewart *et al*, 1958) and a Scottish case–control study (Kinlen *et al*, 1993a). Cases were defined as children registered with the NRCT who were born in the UK between 1952 and 1986 inclusive and who were diagnosed, also between these years, as having a malignant neoplasm or brain tumour before the age of 15 years. A total of 35 949 cases matching the study entry criteria were identified. Parental data were obtained on the fathers of 34 538 cases and the mothers of 35 648 cases. Individually matched controls were selected as described previously (Draper *et al*, 1997b).

Record linkage techniques were used to compare the details of the parents of both cases and controls with those of radiation workers held on the NRRW (Kendall *et al*, 1992; Muirhead *et al*, 1999) to identify those parents who were monitored radiation workers before the conception of the child. Conception was assumed to occur 270 days prior to the birth of a child. A total of 161 fathers and 18 mothers who were monitored prior to conception of the relevant child were identified. Cases and controls whose fathers or mothers were only monitored after conception of the child in question were not included in the study.

### New dosimetric data

The dosimetric data used in the original analyses were based on records in the NRRW, augmented by more details of dose timing. Such data include corrections to allow for dose record keeping practices (Draper *et al*, 1997b). For workers who were also in the Nuclear Industry Combined Epidemiological Analysis (NICEA) (Carpenter *et al*, 1994), more detailed corrected dose information has now become available. Additional dose data for some former workers at British Energy and Magnox Generation have also been obtained.

The new dose information changed the corrected total preconception dose for 67 of the 161 linked fathers. The 6- and 3-month corrected preconception doses changed for 41 and 37 linked fathers, respectively. The total preconception dose changed for 12 of the 18 linked mothers but the 6- and 3-month preconception doses only changed for a single individual. In all but a few instances the dose had increased. These revisions can be attributed mainly to the replacement of zero doses, recorded where the true measurement fell below a predefined recording level, with doses estimated from the distribution of other above-threshold values obtained from the same worker at different times (Carpenter *et al*, 1994).

The analysis of timing of paternal employment contains an extra two linked cases that were excluded from the dose analyses due to incompleteness of dosimetric information.

## ANALYSES

The analysis relating to paternal timing of employment followed the statistical procedures used in the original study. Three binary variables were defined: namely, left employment before the date of conception, employed on the date of conception and employed in the year of diagnosis. First, in separate analyses, a relative risk (RR) and associated 95% CI was calculated for each of these variables. Then the RRs and CIs were recalculated in a simultaneous analysis of the three variables. This enabled the independent effects of any of these variables to be assessed. For all the models fitted, the significance of these variables, as compared to the null model, was tested using the likelihood ratio test. Radiation work carried out after the date of diagnosis for a case child (or the corresponding date for a control child) was ignored.

The analyses and model fitting, both continuous and categorical, carried out previously on the total corrected preconception dose and the 3- and 6-month corrected preconception dose data were repeated using the revised dosimetry data. Exact methods (LogXact, 1993) were used to analyse dose as a categorical variable. Log-linear conditional logistic regression models were employed to analyse dose as a continuous variable, using PECAN (Preston *et al*, 1998). Both analyses are based on conditional likelihoods appropriate for matched case-control data (Breslow and Day 1980; McCullagh and Nelder, 1989; see Draper *et al*, 1997b for further details). Results were considered to be statistically significant if a two-sided *P*-value of less than 0.05 was obtained.

## RESULTS

### Timing of paternal employment

The results concerning the timing of paternal employment are summarised in Table 1

**Table 1:** Relative risks by time of paternal employment at facilities participating in the National Registry for Radiation Workers

			**Separate analyses of the three variables**		**Simultaneous analysis of the three variables**	
**Variable with levels**	**Cases**	**Controls**	**RR**	**(95% CI)^a^**	**RR**	**(95% CI)^a^**
**Leukaemia and non-Hodgkin's lymphoma**						
*Left employment before conception*^b^						
No	13 637	16 008	1.0			
Yes	12	15	1.04	(0.46–2.35)	1.08	(0.47–2.46)
*Employed on date of conception*^b^						
No	13 612	15 994	1.0			
Yes	37	29	2.34**	(1.31–4.18)	2.13	(0.66–6.79)
*Employed in year of diagnosis*^b^,^c^						
No	13 617	15 999	1.0			
Yes	32	24	2.26**	(1.22–4.19)	1.13	(0.33–3.89)
						
**Cancers other than leukaemia and non-Hodgkin's lymphoma**						
*Left employment before conception*^b^						
No	20 877	20 875	1.0			
Yes	12	14	0.85	(0.38–1.89)	0.85	(0.38–1.89)
*Employed on date of conception*^b^						
No	20 868	20 866	1.0			
Yes	21	23	0.91	(0.51–1.65)	0.99	(0.32–3.07)
*Employed in year of diagnosis*^b^,^c^						
No	20 874	20 872	1.0			
Yes	15	17	0.88	(0.44–1.77)	0.89	(0.24–3.38)
						
**All childhood cancers**						
*Left employment before conception*^b^						
No	34 514	36 883	1.0			
Yes	24	29	0.94	(0.53–1.66)	0.95	(0.53–1.69)
*Employed on date of conception*^b^						
No	34 480	36 860	1.0			
Yes	58	52	1.49	(0.99–2.25)	1.35	(0.60–3.05)
*Employed in year of diagnosis*^b^,^c^						
No	34 491	36 871	1.0			
Yes	47	41	1.51	(0.96–2.38)	1.13	(0.46–2.80)

^*^P<0.05

** *P*<0.01.

a Exact 95% CI, calculated using LogXact (1993).

b With a nonzero preconceptional radiation dose recorded with the NRRW.

c Refers to 1 January in the year in which the case child was diagnosed.

. These analyses use all available data, including those linked cases previously identified by Gardner *et al* (1990). For LNHL, statistically significantly raised RRs were found in relation to paternal employment on the date of conception (RR 2.34, 95% CI 1.31, 4.18) and for paternal employment on the date of diagnosis (RR 2.26, 95% CI 1.22, 4.19), when these variables were analysed separately. In contrast, the RR for employment that ceased before conception was close to one (RR 1.04, 95% CI 0.46, 2.35). The simultaneous analysis of all these variables did not yield significantly raised risks for any one of the variables. Table 2

**Table 2:** Deviances for models fitted in Table 1

**Variables**	**Deviance (df)**	**LRS^a^**	***P***
*Leukaemia and non-Hodgkin's lymphoma*			
Left employment before conception^b^	149.71 (1)	0.011	0.92
Employed on date of conception^b^	141.12 (1)	8.60	0.003
Employed in year of diagnosis^b^,^c^	142.65 (1)	7.07	0.008
Simultaneous analysis^d^	141.04 (3)	8.68	0.03
			
*Cancers other than leukaemia and non-Hodgkin's lymphoma*			
Left employment before conception^b^	106.58 (1)	0.17	0.68
Employed on date of conception^b^	106.65 (1)	0.09	0.76
Employed in year of diagnosis^b^,^c^	106.62 (1)	0.13	0.72
Simultaneous analysis^d^	106.45 (3)	0.29	0.96
			
*All childhood cancers*			
Left employment before conception^b^	256.42 (1)	0.048	0.83
Employed on date of conception^b^	252.78 (1)	3.69	0.055
Employed in year of diagnosis^b^,^c^	253.28 (1)	3.19	0.074
Simultaneous analysis^d^	252.68 (3)	3.78	0.29

a Likelihood ratio statistic.

b With a nonzero preconceptional radiation dose recorded with the NRRW.

c Refers to 1 January in the year in which the case child was diagnosed.

d Based on fitting all three variables.

details the deviances of the models and tests of statistical significance based on the likelihood ratio statistic. For LNHL, most of the deviance reduction of the simultaneous analysis can be explained either by the variable employed on the date of conception or by the variable employed in the year of diagnosis.

Additional analyses of LNHL were carried out restricting the data to those workers whose employment data were supplied by NICEA, that is, using what are thought to be the most reliable data. This raised the RR slightly for the variables ‘employed on date of conception’ and ‘employed in year of diagnosis’ to 2.70 (95% CI 1.11, 6.58) and 3.00 (95% CI 1.22, 7.37) for the separate analyses.

### New dosimetric analyses

#### Paternal dose

The results of the categorical analysis of preconception dose, excluding the Gardner cases and their controls, are given in Table 3

**Table 3:** Relative risks for childhood cancer and paternal preconception dose categories: updated national data set excluding for LNHL ‘Gardner cases’ and their controls

**Variable**	**Dose group (mSv)**	**Cases (number in previous study)**	**Controls (number in previous study)**	**Relative risk**	**95% CI^b^**
*Leukaemia and non-Hodgkin's lymphoma (13 621 cases, 15 995 controls)*					
Nonradiation worker^c^		13 581	15 957	1.0	
Total preconception dose	<0.1	5 (6)	0 (0)	6.73^d^	(0.92–∞)
	0.1–49.9	30 (29)	32 (32)	1.53	(0.85–2.77)
	50.0–99.9	3 (4)	2 (2)	3.83	(0.42–47.67)
	100.0+	2 (1)	4 (4)	0.86	(0.07–6.57)
					
6 months preconception dose	<0.1^e^	19 (20)	17 (19)	1.60	(0.75–3.44)
	0.1–4.9	18 (17)	16 (14)	2.10	(0.93–4.91)
	5.0–9.9	0 (1)	1 (1)	3.00^d^	(0.00–117.00)
	10.0+	3 (2)	4 (4)	1.82	(0.22–14.83)
					
3 months preconception dose	<0.1^e^	19 (22)	21 (22)	1.31	(0.64–2.68)
	0.1–2.4	18 (16)	12 (11)	3.21	(1.29–8.79)
	2.5–4.9	0 (0)	1 (2)	1.00^d^	(0.00–39.00)
	5.0+	3 (2)	4 (3)	1.88	(0.23–15.63)
					
*All cancers excluding leukaemia and non-Hodgkin's lymphoma (20 889 cases, 20 889 controls)*					
Nonradiation worker^c^		20 856	20 854	1.0	
Total preconception dose	<0.1	1 (1)	1 (2)	1.00	(0.01–78.50)
	0.1–49.9	27 (27)	29 (28)	0.93	(0.52–1.64)
	50.0–99.9	3 (3)	3 (3)	0.99	(0.13–7.38)
	100.0+	2 (2)	2 (2)	1.00	(0.07–13.80)
					
*All cancers (34 510 cases, 36 884 controls)*					
Nonradiation worker^c^		34 437	36 811	1.0	
Total preconception dose	<0.1	6 (7)	1 (2)	6.00	(0.73–276.00)
	0.1–49.9	57 (56)	61 (60)	1.19	(0.79–1.78)
	50.0–99.9	6 (7)	5 (5)	1.84	(0.45–8.00)
	100.0+	4 (9)	6 (6)	0.91	(0.18–4.01)

a Leukaemia and nonHodgkin's lymphoma cases (and associated controls) born and diagnosed in the West Cumbria Health District in the years 1952–1985.

b Exact 95% confidence interval, calculated using LogXact (1993).

c No radiation dose recorded with the NRRW before conception of the survey child. All relative risks are calculated using this as the reference group.

d Conditional maximum-likelihood estimate is not available because the sufficient statistic is at one extreme of its range. The median unbiased point estimate is shown.

e Includes members of the NRRW who only had radiation doses in earlier time periods.

. There were some minor changes, illustrated in the table, in the distribution of cases and controls among the dose groups. In the original categorical analysis (Draper *et al*, 1997a,Draper *et al*, 1997b), only two of the RRs were found to be statistically significantly elevated. The first was for LNHL and for the lowest exposed category of total preconception dose (<0.1 mSv) (six cases and zero control fathers, RR 8.17, 95% CI 1.18, ∞). The second was for LNHL for the 0.1–2.4 mSv dose group, for doses received in the 3 months prior to conception (16 cases and 11 control fathers, RR 2.82, 95% CI 1.10, 7.82). In the current analysis, the first of these risks was reduced to 6.73 (95% CI 0.92, ∞) and was now no longer significantly different from one. The second result was changed little, with an RR of 3.21 (95% CI 1.29, 8.79).

The relative risk for LNHL associated with a total preconception dose of 100 mSv or more was found in the Gardner study (1992) to be significantly raised at 6.45 (95% CI 1.57, 26.48). In our original study (Draper *et al*, 1997a), the corresponding RR was found to be 0.46 (95% CI 0.01, 5.17), based on one case and four control fathers. In this analysis, a value of 0.86 (95% CI 0.07, 6.57) based on two cases and four control fathers was found.

The analyses using dose as a continuous variable were designed to evaluate possible trends in risk, again excluding the Gardner cases and controls. There were only minor differences in the results of these analyses when they were repeated using the revised dose data, as shown in Table 4

**Table 4:** Relative risk of childhood cancer for specified paternal dose variables: updated national dataset excluding for LNHL ‘Gardner cases’ and their controls^a^

		**Leukaemia and NHL**		**All cancers**	
**Model^b^**	**Adjustment^c^**	**RR^d^**	**95% CI^d^**	**RR^d^**	**95% CI^d^**
*100 mSv cumulative paternal preconception dose*					
Exponential	Unadjusted	1.65	(0.64–4.62)	1.41	(0.67–3.09)
Exponential	Adjusted	0.96	(0.31–2.93)	1.10	(0.46–2.68)
Linear	Unadjusted	2.54	(0.73–8.47)	1.58	(0.72–3.91)
					
*10 mSv 6-month paternal preconception dose*					
Exponential	Unadjusted	1.77	(0.57–6.00)	1.49	(0.73–3.27)
Exponential	Adjusted	1.04	(0.28–3.77)	1.26	(0.58–2.90)
					
*5 mSv 3-month paternal preconception dose*					
Exponential	Unadjusted	1.46	(0.40–5.59)	1.34	(0.66–2.92)
Exponential	Adjusted	0.75	(0.17–3.16)	1.11	(0.52–2.53)
					
*100 mSv paternal post-natal dose*^e^					
Exponential	Unadjusted	1.07	(0.09–11.53)	2.72	(0.76–14.10)
Exponential	Adjusted	0.28	(0.01–3.74)	2.09	(0.55–11.54)

a Leukaemia and non-Hodgkin's lymphoma cases (and associated controls) born and diagnosed in the West Cumbria Health District in the years 1952–1985.

b Exposure treated as continuous variable.

c Adjusted fits incorporate allowance for the effects of the father being a radiation worker. Results based on a linear adjusted model are not shown as they are very similar to those for the exponential adjusted model.

d Calculated using conditional logistic regression in PECAN (Preston *et al*, 1998).

e All analyses including those for postnatal doses relate only to individuals with preconception doses.

. For example, the relative risk of LNHL associated with a 100 mSv cumulative paternal preconception dose, based on the exponential model with adjustment for the effects of being a radiation worker, was 0.96 (95% CI 0.31, 2.93) here, compared with 0.92 (95% CI 0.28, 2.98) in the original analysis. Again, the results for the linear model with adjustment are very similar to those for the exponential model with adjustment.

#### Maternal dose

The revised distribution of cases and controls among the maternal preconceptional dose categories is given in Table 5

**Table 5:** Numbers of childhood cancer cases and controls by maternal preconception dose categories

**Variable**	**Dose group (mSv)**	**Cases (number in previous study)**	**Controls (number in previous study)**
*Leukaemia and non-Hodgkin's lymphoma (13 859 cases, 13 859 controls)*			
Nonradiation worker^a^		13 855	13 858
Total preconception dose	<0.1	0 (0)	0 (0)
	0.1–4.9	3 (3)	1 (1)
	5.0–49.9	0 (1)	0 (0)
	50.0+	1 (0)	0 (0)
			
			
*All cancers excluding leukaemia and non-Hodgkin's lymphoma (21 789 cases, 21 789 controls)*			
Nonradiation worker^a^		21 778	21 787
Total preconception dose	<0.1	2 (2)	0 (0)
	0.1–4.9	5 (6)	1 (2)
	5.0–49.9	2 (2)	1 (0)
	50.0+	2 (1)	0 (0)
			
*All cancers (35 648 cases, 35 648 controls)*			
Nonradiation worker^a^		35 633	35 645
Total preconception dose	<0.1	2 (2)	0 (0)
	0.1–4.9	8 (9)	2 (3)
	5.0–49.9	2 (3)	1 (0)
	50.0+	3 (1)	0 (0)

a No radiation dose recorded with the NRRW before the conception of the survey child.

; other parts of the maternal analysis were unchanged. A small number of cases and controls changed dose groups, but the overall picture was similar to before. RRs are not presented as the number of linked mothers is so small.

## DISCUSSION

Our original study found that fathers of children with LNHL were more likely to be radiation workers than fathers of controls. The present analysis of the timing of paternal employment revealed that the increased risk is concentrated among those children whose fathers were radiation workers either at the time of conception or diagnosis, and not among children whose fathers who left radiation work before conception. It was not possible to determine which of the first two employment variables was the more important, because the number of affected children was small, radiation work at conception and diagnosis were highly correlated, and data were not available for linkages to fathers who were monitored only after conception.

A reanalysis was also undertaken to determine the effects of changes to the dosimetric data on the original conclusions. As before, there was no positive association of cancer risk with parental preconception dose. The results of the maternal analyses are essentially unchanged: mothers of children with cancer were more likely to be radiation workers than mothers of controls, but the association was not related to radiation dose.

There is evidence that childhood leukaemia rates are increased in areas of marked rural population mixing, that is, when many individuals from different areas move into a rural area, where susceptible individuals are more prevalent (Kinlen, 1995; Kinlen and Petridou, 1995; Kinlen *et al*, 1995; Stiller and Boyle, 1996; Dickinson and Parker, 1999; Kinlen and Balkwill, 2001). Such influxes would increase population density and hence the level of contacts between susceptible and infected individuals, thereby promoting epidemics of this (mainly subclinical) infection. This is consistent with the hypothesis that childhood leukaemia is a rare response to a common but unidentified infection.

Certain studies of population mixing have pointed to adult transmission of infection in the aetiology of childhood LNHL (Kinlen *et al*, 1993b,Kinlen *et al*, 1995; Kinlen, 1995). The nuclear industry is unusual in the siting of installations in rural and even isolated areas, the extent to which it brings many people together from different parts of the country, and the extent to which there is movement among different installations. Thus, radiation workers may be subject to above-average levels of population mixing and hence of exposure to oncogenic infections. Furthermore, the localities of certain nuclear sites (notably Dounreay and Aldermaston) have been exposed to unusual levels of population mixing for reasons unconnected with radiation work (Kinlen, 1995). Our new findings, though limited, are consistent with the hypothesis that transmission of oncogenic infection from parents takes place in early life. As before, our results provide no support for the PPI hypothesis.
